# Transcending differences to study the transcendent: an exploratory study of researchers’ and chaplains’ reflections on interdisciplinary spiritual care research collaboration

**DOI:** 10.1186/s12904-015-0004-4

**Published:** 2015-04-18

**Authors:** Richard A Powell, Linda Emanuel, George Handzo, John Lantos, Laura B Dunn, Ellen L Idler, Diane J Wilkie, Kevin Massey, William T Summerfelt, Marilyn JD Barnes, Tammie E Quest, Allison Kestenbaum, Karen Steinhauser, George Fitchett, Angelika Zollfrank, Annette K Olsen, Tracy A Balboni, Dane Sommer

**Affiliations:** Research, HealthCare Chaplaincy Network, New York, USA; Global Health Researcher, Nairobi, Kenya; Research and Education, HealthCare Chaplaincy Network, 65 Broadway, 12th Floor, New York, NY 10006-2503 USA; Geriatric Medicine and Buehler Center on Aging, Health & Society, Feinberg School of Medicine, Northwestern University, Chicago, USA; Health Services, Research and Quality, HealthCare Chaplaincy Network, New York, USA; Pediatrics, Children’s Mercy Hospital and University of Missouri at Kansas City, Kansas City, Missouri USA; Psychiatry, Department of Psychiatry, UCSF Helen Diller Family Comprehensive Cancer Center, University of California, San Francisco, USA; Emory University, Atlanta, Georgia USA; Center of Excellence for End-of-Life Transition Research, Department of Biobehavioral Health Science, College of Nursing, University of Illinois at Chicago, Illinois, USA; Mission and Spiritual Care, Advocate Lutheran General Hospital, Chicago, Illinois USA; Advocate Health Care, Downers Grove, Chicago, Illinois USA; Department of Mission and Spiritual Care, Advocate Lutheran General Hospital, Chicago, Illinois USA; Emory Palliative Care Center, Emory University, Atlanta, Georgia USA; Association of Clinical Pastoral Education, Center for Pastoral Education, The Jewish Theological Seminary, New York, USA; Department of Medicine, Duke University, North Carolina, USA; Department of Religion, Health and Human Values, Rush University Medical Center, College of Health Sciences, Rush University, Chicago, Illinois USA; Clinical Pastoral Education, Yale-New Haven Hospital, New Haven, CT USA; Clinical Administrative Chaplain, Duke University Medical Center, Durham, North Carolina USA; Departments of Radiation Oncology, Dana-Farber/Brigham and Women’s Cancer Center, Harvard Medical School, Boston, USA; Chaplaincy Services, Children’s Mercy Hospital, Kansas City, Missouri, USA

**Keywords:** Spiritual, Care, Biomedical, Interdisciplinary, Collaboration

## Abstract

**Background:**

Despite recognition of the centrality of professional board-certified chaplains (BCC) in palliative care, the discipline has little research to guide its practices. To help address this limitation, HealthCare Chaplaincy Network funded six proposals in which BCCs worked collaboratively with established researchers. Recognizing the importance of interdisciplinary collaboration in the development of a new field, this paper reports on an exploratory study of project members’ reflections over time on the benefits and challenges of conducting inter-disciplinary spiritual care research.

**Methods:**

Data collection occurred in two stages. *Stage 1* entailed two independent, self-reflective focus groups, organized by professional discipline, mid-way through the site projects. *Stage 2* entailed end-of-project site reports and a conference questionnaire.

**Results:**

Eighteen professionals participated in the group discussions. *Stage 1*: researchers perceived chaplains as eager workers passionately committed to their patients and to research, and identified challenges faced by chaplains in learning to conduct research. Chaplains perceived researchers as passionate about their work, were concerned research might uncover negative findings for their profession, and sensed they used a dissimilar paradigm from their research colleagues regarding the ‘ways of relating’ to knowledge and understanding.

*Stage 2*: researchers and chaplains noted important changes they ascribed to the interdisciplinary collaboration that were classified into six domains of cultural and philosophical understanding: respect; learning; discovery; creativity; fruitful partnerships; and learning needs.

**Conclusions:**

Chaplains and researchers initially expressed divergent perspectives on the research collaborations. During the projects’ lifespans, these differences were acknowledged and addressed. Mutual appreciation for each discipline’s strengths and contributions to inter-professional dialogue emerged.

## Background

Spiritual care is recognized as an essential component of quality palliative care [1-3]. In the USA, spiritual care needs are often addressed by professional, board-certified chaplains (BCCs); however, few rigorous studies exist describing or evaluating chaplaincy services in the palliative care setting [4,5]. Chaplaincy research has only recently begun to employ methodologically rigorous study designs [6], develop a body of outcomes data and other knowledge to inform practice [7], and focus on nurturing a community of chaplain-researchers.

To address some of these limitations, in 2011 HealthCare Chaplaincy Network (HCCN) started work to foster a sustainable, critical mass of chaplain-researchers by engaging them in research that addressed substantive spiritual care questions. HCCN sought proposals in which BCCs worked collaboratively with established biomedical or behavioral science researchers and funded six projects (see Table 1) that collectively, along with the faculty-advisors to the projects, formed the founding membership of the Chaplaincy Research Collaborative (CRC).

**Table 1:** **Funded studies of the Chaplaincy Research Collaborative**

**Study title**	**Site**
Hospital chaplaincy and medical outcomes at the end of life	Dana Farber Cancer Institute, Harvard University, Massachusetts
Spiritual assessment and intervention model (AIM) in outpatient palliative care for patients with advanced cancer	University of California, San Francisco, California
Understanding pediatric chaplaincy in crisis situations	Children’s Mercy Hospital, Missouri
‘What do I do’ – developing a taxonomy of chaplaincy activities and interventions for spiritual care in ICU palliative care	Advocate Charitable Foundation & Advocate Health Care (Chicago), Illinois
Impact of hospital-based chaplain support on decision-making during serious illness in a diverse urban palliative care population	Emory University, Georgia
Caregiver outlook: an evidence-based intervention for the chaplain toolkit	Duke University Medical Center, North Carolina

Collaboration across – as well as within – disciplines can be problematic. Distinct cultural values, norms, processes, paradigms, communication methods and organizational barriers can result in inter-disciplinary discord, and ‘tribal’ alliances that negatively impact the patient experience [8,9]. Social science has long accepted that before they can deliver high quality results, such team-based collaborations go through a phased development process. The classic articulation of these functioning growth stages was by Tuckman in 1965 following his analysis of team dynamics, and entailed: Forming (the initial, pre-project preparatory phase) – Storming (the phase of initial team collaboration often characterized by individual competition for status and ideas’ acceptance, with conflict that is addressed by learning how to solve problems together) – Norming (when the team begins to work more effectively) and – Performing (when the team is functioning at a very high level, with a focus on reaching goals as a group) [10]. In this model, all four stages are necessary and inevitable to develop, address challenges, tackle problems, find solutions, plan work, and deliver results. Contributing to a nascent field in which inter-professional collaboration will be critical to its success, this paper reports on an exploratory study of CRC team members’ reflections over time on the benefits and challenges of inter-disciplinary spiritual care research.

## Methods

### Sample

Respondents included all CRC members, both biomedical or behavioral science researchers and BCCs, participating in and advising the six funded projects. Written informed consent to participate in the study was obtained from all participants. Given the exploratory and preliminary nature of this project in a very new field, all participants were offered the opportunity to also contribute to the writing of the report.

### Data collection

This occurred over two stages. *Stage* 1: two independent, self-reflective focus groups, separated by professional discipline (i.e., BCCs and biomedical/behavioral science researchers), and conducted at a CRC symposium mid-way through site projects (June 2013). Participants completed a brief form providing socio-demographic and research experience data and responded to three questions:

“What has your experience been working with chaplains/researchers in your team?”

“What do you think could make things work better?”

“What are some of the professional characteristics that compare and contrast between chaplaincy and researcher cultures?”

Discussions were facilitated by an experienced chaplain (GH) and palliative care researcher (LE), who were also the co-principal investigators for the project. Identified themes were recorded on flipcharts.

*Stage 2*: CRC members were asked to reflect on two questions in end-of-project reports (January 2014):

“What do you consider the benefits and potential drawbacks of working collaboratively in support of the aims of this project?”

“Share with us the thoughts of your research team on how those interested in this area of research, and the inclusion of chaplains in those projects, might continue to work together collaboratively to attain these aims?”

CRC members were asked to provide additional feedback in response to a brief questionnaire at the end-of-project conference (March 2014).

### Data analysis

In Stage 1, profiling socio-demographic and profession data were entered into an Excel spreadsheet for descriptive analysis and presented as simple frequencies. In view of the near-verbatim nature of the data recorded directly onto the flipcharts, the authors decided against formal qualitative analysis, instead reviewing and categorizing the topics into thematic groups. Agreement with the final categories, and their validation, was obtained from all participants. Given the number of participants was small, the authors presented the data by professional group and site only, rather than by any other potentially contextualizing factor (e.g., gender, age), with the quotes cited as summarizing group perspectives rather than attributable to individuals.

In Stage 2, CRC members’ statements were collated and emergent themes recorded by two authors (LE and GH), with disagreements – which were minimal in nature – resolved by consensus discussion.

### Ethics

The study was deemed exempt locally from institutional board review by the Children's Mercy Hospital, Missouri.

## Results

### Stage 1

Participants’ profiles from Stage 1 are outlined in Table 2.

**Table 2:** **Socio-demographic and research characteristics of Stage 1 participants**

**Variable**	**N**
*Profession*	
Chaplaincy	8
Non-chaplaincy	10
*Gender*	
Male	8
Female	10
*Age*	
20-39	3
40-49	6
50-59	6
60+	3
Median yrs in research*	
Chaplaincy	6.5 (range: 1–25)
Non-chaplaincy	18 (range: 8–36)
Median yrs in spiritual care research*	
Chaplaincy	1 (range: 0–25)
Non-chaplaincy	5.5 (range: 1–30)

Note: *One of the BCC-trained participants also had a significantly long research career.

#### (i) Characteristics of participants

Eighteen professionals participated in the group discussions.

There were slightly more non-chaplains (10 versus 8) – with researchers representing sociology, psychiatry, nursing, psychology, medicine, psycho-oncology and palliative care – and more women (10 versus 8). Non-chaplains had been involved in research generally for significantly longer than BCCs (18 versus 6.5 years).

#### (ii) Perceptions of professional characteristics and experiences

##### Researchers

Researchers perceived chaplains as easy to work with, seeing them as eager workers passionately committed to their patients and research. When asked to think of words describing chaplains, they used numerous highly complementary terms – e.g., thoughtful; enthusiastic; reflective; hard working; deep, as well as systematic thinkers.

The researchers identified challenges chaplains faced in participating effectively in a research team. These included technological and logistical issues (e.g., dealing with information technology, databases, and audio recordings). Additionally, researchers perceived that chaplains were generally untrained in research.

Researchers also perceived that some chaplains experienced research as a threat to their profession, e.g., feeling they were being judged. Other researchers noted some chaplains’ concerns about the potential impact of being observed on the effectiveness of their chaplaincy practice.

Another common theme noted by researchers was some chaplains’ apparent deep-rooted questioning of the appropriateness and effectiveness of research in studying issues previously considered by some as elusive, unquantifiable or sacred, exemplified by researchers following perceptions:

“You can’t measure the divine”;

“Don’t unpack the ‘special”, and;

“Sacred means don’t probe”.

##### Chaplains

Chaplains perceived researchers as “passionate about what they do.” They also expressed a “fear of being exposed”; specifically, that research studies might uncover negative findings, e.g., demonstrating that pastoral interventions are ineffective and thereby harming chaplaincy as a profession.

Chaplains reported feeling their research skill sets were inadequate. They felt basic training should be a prerequisite for effective research participation. However, they also reported experiencing unrealistic expectations from their research colleagues regarding their level of research training and education.

Chaplains suggested they used a dissimilar paradigm from their research colleagues in terms of their “ways of relating” to knowledge and understanding. As one commented:

“Chaplains and researchers talk differently. A researcher may ask, ‘How do you know that?’ ‘What’s your data?’ Whereas a chaplain may say, ‘I know that by my experience …’ and … chaplains perceive [that] as the disconnect between art and science”.

#### (iii) Improving collaboration

##### Researchers

In addition to understanding chaplains’ concerns regarding research collaboration, researchers acknowledged the need to identify chaplains as “the experts” in the spiritual domain, and to convey their appreciation of that fact. Additionally, creating learning opportunities – e.g., “How database people and chaplains (work) together” – was important, as was fostering an inclusive inter-disciplinary team atmosphere by having such events as a “Series of ‘lunch and learns’”.

##### Chaplains

Chaplains wanted researchers to have a more realistic appreciation of their existing research abilities (e.g., evaluating ability with a project management checklist). Additionally, it was considered critical that directors of chaplaincy care services are fully supportive of the chaplains’ participation in research – including an accurate appreciation of its time commitments – with transparency regarding the multiple work accountabilities involved.

Lastly, there was an emphasis accorded to collaboration as a way forward, by ‘Keep(ing) making an intentional effort to stay together” in a supportive, equitable partnership: “Established as mutuality – two-way learning”.

### Stage 2

Primary findings from end-of-project reports are presented thematically (see Table 3). Two main challenges emerged: researchers perceived chaplains lacked knowledge of basic research principles, rendering it difficult for chaplains to be optimal collaborators. To help address this challenge, researchers suggested that “Future projects need to include a supplemental research training component and/or time funded for mentorship” (SD)^a^. Second, there was a general BCC reflection regarding “the (severity of the) learning curve associated with the deepening of the professional relationships” (MA).

**Table 3:** ** List of end-of-project reflections on the benefits of, and lessons learnt from, collaboration**

**Theme 1: Respect**
“We knew at the outset that researchers and chaplains had different agendas, goals, and interests. We anticipated some tensions as we went. We were surprised, though, that the tensions that developed were not usually the ones we anticipated. While the chaplains were somewhat reticent to participate in research, it was NOT because they questioned the value of research or thought that their work was so ineffable that it could not be studied. Instead, their concerns were about the risks of research to the patients. Those risks were not the typical risks of biomedical research (i.e. the risks of an experimental drug or innovative procedure.) Instead, they were the risks that might arise from the effect of research on the chaplains’ own work with families. They feared that, in being observed, they might not do their jobs as well.” (LC)
“We couldn’t have done our project without the enthusiastic participation of the chaplains. The chaplains helped us make the project doable by giving valuable feedback on every aspect of our study design and methodology.” (LC)
“Take some time to get to know you colleagues and their perspectives. Respect all of the individual contributors to the team and praise each other for small wins. Learn about the culture of chaplains, how chaplains are training and how different this might be compared to other members of the team.” (QE)
“The benefits of working collaboratively … are that both chaplains and researcher grew in appreciation of each other’s contribution. In our project, both disciplines had little or no contact prior to the project and now are envisioning numerous future collaborations.” (MA)
**Theme 2: Learning**
“(This was) an opportunity for chaplains to educate researchers and clinicians in areas considered importance to chaplains.” (SD)
“In virtually every team meeting the chaplains, experienced researchers, or both were able to lend their unique perspective to a common problem or question. For example, when writing our time diaries, we needed the input of our chaplain team members …” (QE)
“The effect of new perspectives was even more pronounced in our Community Advisory Board meetings, where patient and family advisors and other practicing chaplains and community ministers never failed to deepen our understanding and strengthen the framework through which we were viewing our data.” (QE)
**Theme 3: Discovery**
“Non-chaplain professional researchers gained new terminology.” (SD)
“The different lexicons of researchers and chaplains presented an opportunity for researchers to learn the language of chaplaincy and further the ability to do the work thoughtfully.” (QE)
“The need to identify, negotiate, discuss roles and role expectations, and understand the different skill set that each brings to the project. Chaplains themselves do not always have a shared understanding of key terms, roles, and boundaries. While variation exists within most disciplinary groups, we were struck by the lower scope of standardization and high variance. Such variance contributes to challenges of communicating chaplains’ skills and recommendations in a consistent or unified way.” (SD)
“Clarify terms and definitions early in project to create a working dictionary to establish boundaries and create shared understanding of key terms, even those as ‘simple’ as ‘spirituality’ and ‘religion.’” (SD)
**Theme 4: Creativity**
“We have found and embraced unique challenges in analyzing data across several disciplines; this led to novel ideas for manuscripts.” (DS)
“Chaplains are excellent sources of study topics and ideas and may very well provide the intellectual … impetus for a study.” (QE)
**Theme 5: Fruitful partnerships**
“Our project included chaplains at every step of the project. Many of the chaplains participated in 3 or more of the research methods associated with out project. The methods team (researchers) and chaplain researchers reviewed results, discussed modifications to the research methods and collectively worked on the publication. Throughout the process, there was not an ‘us-and-them’ mentality. The process was a partnership toward one collective goal.” (MA)
“Our experienced researchers feel we would not have been able to create ‘field-advancing research’ that could be communicated effectively to the chaplain community without chaplain involvement on the project team. Similarly, our chaplains report they would not have been able to launch and carry through such a complex project without the help of experienced researchers.” (QE)
“The research is a collaborative effort with each member, chaplain and researcher, bringing their skills to the table. Learning occurs for the researcher and the chaplain within this partnership and the vocation of chaplaincy benefits, which maps to enhanced spiritual outcomes for patients, family members and staff.” (MA)
**Theme 6: Learning needs**
“One potential drawback to working collaboratively in this particular way in support of the goals of the project is that amidst the busy clinical schedule of BCCs, there is not enough time or resources to provide a complete and in-depth training on research methodology … This is why we hope … to provide funding for chaplain-researchers to complete a training program on empirical research methods, in order to enable them to more deeply and more fully understand empirical research.” (BH)
“Assess level of mentoring chaplain may benefit from and build into project from the beginning.” (SD)

Six primary themes were identified in the collaborative process: respect; learning; discovery; creativity; fruitful partnerships; and learning needs. While participants provided positive overall evaluation of their collaborations, they also identified an important need to clarify the critical terms and definitions of the lexicon that underpinned the new collaborations – including regarding ostensibly basic terminology.

At the end-of-project conference, feedback from the group indicated:

- All researchers but one felt they had achieved career advancement from their participation, while just under half the chaplains felt they had.
- All groups reported a substantial number of projects and presentations made and proposed emanating from their work, reflecting feasible career possibilities.
- Researchers planned more project proposals and publications than chaplains (range 2–5 for researchers versus 2–3 for chaplains, 5–8 versus 2- > 5, respectively) but a similar numbers of presentations (5–7 for researchers versus 4–10 for chaplains).

## Discussion and conclusions

This exploratory study has limitations, as it was conducted among a convenience sample of researchers and BCCs in a fledgling learning partnership aiming to identify some of the pertinent issues involved in inter-disciplinary spiritual care research. Consequently, the conclusions derived are circumspect.

However, it has highlighted a number of interesting provisional findings. Chaplains and researchers initially expressed varying ways of seeing the world. Chaplains’ concerns about the appropriateness and effects of researching the spiritual domain appeared to reflect both their personal beliefs and their relative unfamiliarity with the culture of research. Researchers also expressed concerns regarding chaplains’ relative research inexperience – part of the *raison d’ être* for this project.

However, and acknowledging the potential impact of different data collection methodologies used in Stages 1 and 2, generally participants appeared to experience positive changes over time in inter-disciplinary cultural and philosophical understanding derived from the collaborations across multiple domains. While the overall benefit from collaboration was experienced among both professional groups, chaplains appeared to be reflecting further on how to build research into their career pathways.

During the projects’ lifespans, initial differences were not only acknowledged but aimed to be addressed with appropriate interventions (e.g., research training for chaplains team members). Moreover, the teams expressed a growing appreciation of each discipline’s strengths and contributions to inter-professional dialogue and functioning. These positive reflections are indicative, along with the list of proposed conference presentations and publications completed and planned from each site, of the ‘performing’ phase of Tuckman’s group dynamics.

The findings underscore that future chaplain-researcher collaboration will require mutual respect, patience, and willingness to reconsider assumptions in both disciplines. For instance, chaplains’ concerns about the applicability of research to the spiritual domain must be respected, not dismissed. Only with increased research experiences will chaplains – and the field of chaplaincy generally – grow more comfortable and confident as professionals who not only provide personalized spiritual care to patients and families, but also conduct research on this care.

Researchers will need to adapt their learning and communications styles to maximize the contributions professional chaplains can make to research, as part of a reciprocal process of accommodation and knowledge acquisition that values professional diversity and is sensitive to the dynamics inherent to professional cultures’ interactions [9].

As for future research arising from this study: this remains a nascent field of enquiry, with limited funding opportunities to conduct larger, more representative studies using a critical mass of chaplain-researchers and established biomedical or behavioral science researchers. However, it is important – and more pragmatic – that existing and future research networks explore further the inter-disciplinary issues identified in this study to help identify lessons learnt and best practices to support a growing body of understanding.

## Endnote

^a^Sources attributed to cited quotations in Stage 2 data are constructed by the initial of the site principal investigator’s surname and the first letter of the lead organization (e.g., ‘Evans’ and ‘Yale University’ would be ‘EY’).

## References

[1] Puchalski C, Ferrell B, Virani R, Otis-Green S, Baird P, Bull J (2009). Improving the quality of spiritual care as a dimension of palliative care: the report of the Consensus Conference. J Palliat Med.

[2] Ferrell B, Connor SR, Cordes A, Dahlin CM, Fine PG, Hutton N (2007). The national agenda for quality palliative care: the national consensus project and the national quality forum. J Pain Symptom Manage.

[3] The Joint Commission. Advancing Effective Communication, Cultural Competence, and Patient- and Family-Centered Care: A Roadmap for Hospitals. Oakbrook Terrace, Illinois; The Joint Commission 2010.

[4] Flannelly KJ, Emanuel LL, Handzo GF, Galek K, Silton NR, Carlson M (2012). A national study of chaplaincy services and end-of-life outcomes. BMC Palliat Care.

[5] Flannelly KJ (2012). Spirituality and chaplaincy in palliative care. J Health Care Chaplain.

[6] Galek K, Flannelly KJ, Jankowski KR, Handzo GF (2011). A methodological analysis of chaplaincy research: 2000–2009. J Health Care Chaplain.

[7] Jankowski KR, Handzo GF, Flannelly KJ (2011). Testing the efficacy of chaplaincy care. J Health Care Chaplain.

[8] Weller J, Boyd M, Cumin D (2014). Teams, tribes and patient safety: overcoming barriers to effective teamwork in healthcare. Postgrad Med.

[9] Reich SM, Reich JA (2006). Cultural competence in interdisciplinary collaborations: a method for respecting diversity in research partnerships. Am J Community Psychol.

[10] Tuckman BW (1965). Developmental sequence in small groups. Psychol Bull.

